# An analysis of the outcome of 2,443 women applying to be donors at a commercial egg bank in the USA

**DOI:** 10.1186/s12958-026-01578-1

**Published:** 2026-07-13

**Authors:** Allan A. Pacey, Emilie Lassen, Guido Pennings, Saghar Kasiri, Anne-Bine Skytte

**Affiliations:** 1 School of Medical Sciences, Faculty of Biology, Medicine and Health, Core Technology Facility, University of Manchester, 46 Grafton Street, Manchester, M13 9NT UK; 2 Cryos International, Vesterbro Torv 3, Aarhus, 8000 Denmark; 3 Department of Philosophy and Moral Science, Bioethics Institute Ghent (BIG), Ghent University, Blandijnberg 2, Gent, B-9000 Belgium

**Keywords:** Gamete donation, Anonymity, Infertility, Female infertility, Cryopreservation

## Abstract

**Background:**

Donated eggs are essential for a range of Medically Assisted Reproduction (MAR) procedures, but relatively little has been written about how donors are recruited. This study examined egg donor recruitment processes at a commercial egg-bank in Florida (USA). It documents what proportion of applicants were ultimately accepted and, if rejected, at what step, whether this was by choice or selection, whether their initial ID-release choice was important, and how this compares to the recruitment of sperm donors.

**Methods:**

Anonymised records of all egg donor applicants in 2018 and 2019 (*n* = 2,443) were examined to determine the number passing through (or lost) at each stage of the recruitment process and ultimately how many had successful egg retrieval and cryopreservation. Statistical analysis was carried out to examine differences between the initial ID-release choice made by egg donor applicants (ID-release vs. non-ID release).

**Results:**

Few applicants (2.5%) were accepted and had eggs frozen for donation. This did not differ between applicants who opted at the outset to be ID-release (2.94%) compared to those who didn’t (2.12%) (X_2_ = 1.682; Df = 1; Z = 1.297; *p* = 0.1947). Most were lost during recruitment because they: (i) did not meet the eligibility criteria at the outset (51.17%); (ii) withdrew, failed to respond, did not attend an appointment, or did not return a questionnaire (26.36%); or (iii) reported a disqualifying health issue or failed a screening test (19.69%). There were no significant differences between the initial ID choice of egg donor candidates and the reason for their loss from the process. This differed from what we know about sperm donor recruitment during the same period at the same clinic. Only two women who were accepted to donate failed to do so because of a poor ovarian response. During recruitment, some egg donors decided to change ID-type and it was more common for them to change from non-ID release to ID release (53.57%) than the other way around (9.09%) (X_2_ = 14.920; Df = 1; Z = 3.863; *p* < 0.0001).

**Conclusion:**

This study demonstrates how challenging egg donor recruitment processes are, with only a small fraction of those who initially apply ultimately being accepted and having samples certified as safe for use in treatment. The initial ID-release choice of egg donor applicants in the USA has no bearing on whether they were finally accepted as donors or not and is unrelated to their reason for rejection from the program.

**Supplementary Information:**

The online version contains supplementary material available at 10.1186/s12958-026-01578-1.

## Introduction

The successful recruitment of egg donors is essential to provide a range of Medically Assisted Reproduction (MAR) procedures. For example, the use of donor eggs can provide reproductive options for women with medical conditions such as primary ovarian insufficiency or advanced maternal age as well as for men without a cis female partner and who wish to start a family using surrogacy. Across Europe, 8.00% and 7.64% of MAR cycles performed in 2018 [1] and 2019 [2] respectively used donated eggs. Whilst historically most cycles have been performed using fresh (unfrozen) eggs, recent advances in vitrification technology have allowed the development of commercial egg banks, like Cryos, which can distribute frozen eggs worldwide [3].

For the recruitment and selection of egg donors, guidance has been provided on the medical and laboratory screening of [4–6] and information provision to [7] prospective donors. However, less has been written about how the recruitment of egg donors works in practice and how successful it might be. A few previous studies have described how between 4% [8] and 39% [9] of women who applied to be egg donors were accepted. However, these data are mostly from egg donation programmes recruiting anonymous (or predominantly anonymous) donors and prior to the widespread introduction of egg freezing.

In a recent study of 11,712 men who had applied to be sperm donors at Cryos in Denmark and the USA [10], it was found that only 3.79% of applicants were accepted as donors and had samples frozen and released for use. Moreover, this was statistically different between donors who opted at the outset to be ID-release (4.70%) compared to those who did not (3.15%). A separate study noted that ID-release sperm donors at Cryos were generally older and more likely to have a partner compared to donors who had chosen to be non-ID release [11]. With this in mind, the present paper aims to examine the egg donor recruitment process at Cryos in the USA and reasoned that the initial ID-release choice of candidate donors may also influence their likelihood of being recruited in the same way we have previously described for sperm donors. In addition, for those donors who were not accepted, we also explore at what step their journey ended either by choice or by selection. We also compare these data to what we know about sperm donor recruitment during the same period (2018 and 2019) and in the same gamete bank [10].

## Materials and methods

Data were obtained from the Cryos International databases on every egg donor applicant in their Florida clinic within the calendar years 2018 and 2019. The data collection was limited to egg donor applicants in the USA, because commercial egg banking was not allowed in Denmark (see [12] for further details).

**Fig. 1:**
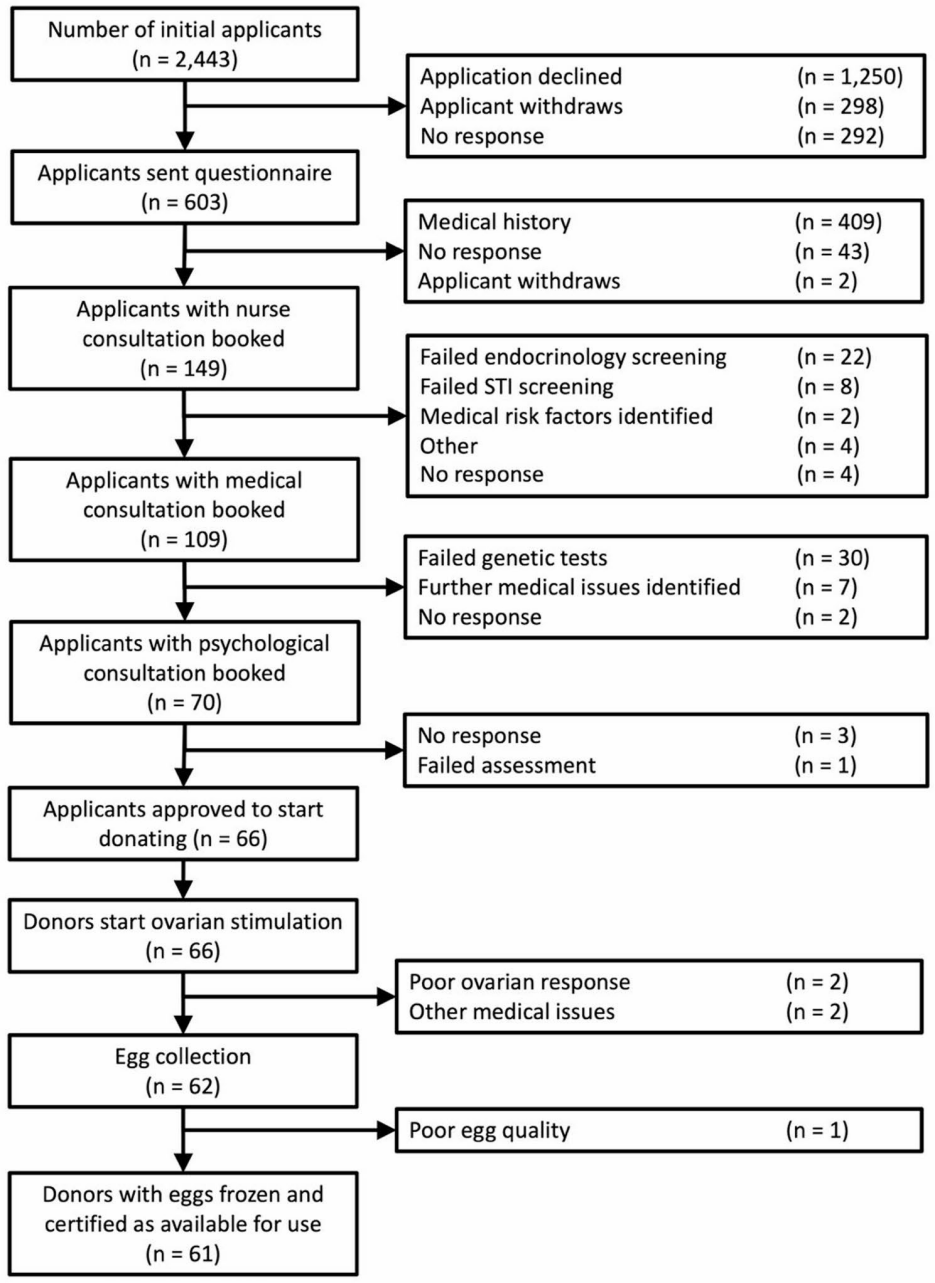
Overview of the stages of egg donor recruitment and the stages at which candidate donors are lost or rejected

The recruitment processes are summarised in Fig. 1 and began when a member of the public filled out an initial online application form available on the Cryos website (www.cryosinternational.com). This included items on basic information (such as age, address) and their initial preference about whether they wanted to be either an ID-release or non-ID release donor, although this decision could be changed later. To be considered as a donor, women had to be between the ages of 18 and 30 years old, with a Body Mass Index (BMI) between 18 and 27 kg/m^2^, be at least 152 cm (5 ft) tall, and be willing to undertake subsequent medical, infectious, genetic, and psychological screening.

Candidate egg donors who passed this stage of the assessment were then sent a detailed medical questionnaire to complete in their own time and return to Cryos either by email or hard copy. This included the following areas: (i) education and profession; (ii) ethnic origin; (iii) questions about health; (iv) questions about infectious diseases and lifestyle; (v) the health of their closest biological family (parents, grandparents, siblings and their children). Upon receipt, the completed medical questionnaire was screened by the donor coordinators / nurses, in close collaboration with reproductive endocrinologists and clinical geneticists. If anything needed to be clarified or documented further, the candidate donor was asked to provide a copy of her medical records from her family doctor.

If the applicant passed this stage, they were invited to a consultation with a nurse where the egg donation process was explained in more detail and the candidate egg donor was asked to give her consent to provide a sample of blood and urine which was used to test for *Chlamydia trachomatis*, *Neisseria gonorrhoeae*, and *Treponema pallidum.* Any applicants who tested positive had their donation deferred for 12 months and were tested again at that point if they wished to continue. The blood sample was also used to check AMH levels and Cryos used a value of ≥ 2.5ng/ml as a cut off below which candidates were not accepted. AMH levels were further considered by a reproductive endocrinologist at a later stage in the process alongside an antral follicle count (AFC) to assess the potential for low ovarian response or rule out polycystic ovarian syndrome. Applicants who did not pass these assessments were rejected and did not receive any financial compensation. All donors who were accepted received a fee per donation of 5,000 USD (for non-ID release) or 5,500 USD (for ID-release) donations respectively. Women could donate up to 6 times, although in this analysis we considered only their first donation.

Egg donor candidates who passed these evaluations were invited to a medical consultation, which included a detailed evaluation of their medical history, as well as a physical examination, and an ultrasound examination to identify any factors which may be a contraindication for ovarian stimulation and egg collection. It was at this stage that applicants gave their consent for genetic screening. The Cryos approach to genetic screening of donors in 2018 and 2019 included testing for 47 recessive disorders as well as Fragile X syndrome (FMR1) [13]. All other screening was performed according to the relevant guidelines published by the European Parliament and the Council of the European Union [4], the Association of Biomedical Andrologists, Association of Clinical Embryologists, British Andrology Society, British Fertility Society, Royal College of Obstetricians & Gynaecologists [14], and the Practice Committee of the American Society for Reproductive Medicine and the Practice Committee of the Society for Assisted Reproductive Technology [5].

Applicants who passed the medical and genetic screening stages were then invited to undertake a psychological assessment by a certified mental health counsellor. This took the format of a consultation lasting up to one hour. No specific assessment tool was used. However, the topics covered were whether the donor was well adjusted socially, their mental health, ability to comprehend the process, identification and discussion of any potential long-term issues, as well as ensuring that the donor candidate fully understood the scope of the donation and the implications of choosing either ID-release or non-ID release status. Only if the psychological assessment was passed did the candidate become approved to start donating and invited to complete consent forms to allow her eggs to be vitrified and donated. Donors completed a range of consent forms depending on which countries she wanted to permit her donations to be used in, and this in-part (along with the number of donations made) determined the number of family groups in which children may be born. Typically, one donation would be used by one (possibly two) recipients. In addition, egg donors provided an extended profile about themselves and were able to change their final ID-release choice up until the day of egg collection. Changes to ID-choice were not permitted beyond this point, but donors were encouraged to maintain contact with the clinic with information about both their own health and the health of their own children (if they had any) and closest family. They were also free to enquire about how successful their donations had been and whether any children had been born.

For this study, data on the number of egg donor applicants passing through (or lost) at each stage of the process were provided to the authors for analysis in an anonymised format. The study was restricted to 2018–2019 to facilitate a direct comparison to the data collected and already published (see [10]) on sperm donor recruitment at the same gamete bank. Statistical analysis was carried out by Chi Squared test, using Graphpad Prism (San Diego, USA) to examine differences between donor type (ID-release vs. non-ID release) at each stage of the recruitment process. Ethical approval for the secondary analysis of anonymised data was granted by the University of Manchester Ethics Committee (Ref: 2023-18751-31889).

## Results

A total of 2,443 women applied via the Cryos website to be considered as an egg donor (Fig. 1) in the study period. Of these, 603 (24.68%) passed the initial online application and were sent a more detailed questionnaire to complete and subsequently 149 (6.10%) had a nurse consultation booked. A total of 109 women then underwent a medical evaluation of which 70 proceeded to a psychological evaluation. The majority of these (66 women) were subsequently approved to start ovarian stimulation to donate eggs, but only 62 (2.54% of initial applicants) underwent egg collection and 61 (2.50% of initial applicants) had eggs frozen and approved for use. Taking all these stages into account, this did not differ between egg donor applicants who opted at the outset to be ID-release (2.94%) compared to those who didn’t (2.12%) (X_2_ = 1.682; Df = 1; Z = 1.297; *p* = 0.1947) (see Supplementary Table 1).

Most candidate egg donors were lost during the recruitment process because they: (i) did not meet the eligibility criteria at the outset and were therefore rejected at the initial screening questionnaire (51.17%); (ii) withdrew, failed to respond, did not attend an appointment or failed to return a questionnaire (26.36%); or (iii) reported a disqualifying health or failed a screening test (19.69%). Only five women who were accepted to donate failed to do so. These were because of a poor ovarian response (*n* = 2), other medical issues (*n* = 2), or poor egg quality (*n* = 1). In each case, there was no obvious relationship between these events and the candidate donor’s initial choice of ID-type (see Supplementary Table 2).

Finally, a total of 18 out of the 61 donors who successfully had eggs frozen decided to change ID-type before their final consent forms were signed and egg collection took place. Overall, it was more common for donors to change from non-ID release to ID release (53.57%) than the other way around (9.09%) (X_2_ = 14.920; Df = 1; Z = 3.863; *p* < 0.0001) although movements in both directions did occur (see Supplementary Table 3).

## Discussion

This study shows that during 2018 and 2019 only 2.50% of the initial applicants to be egg donors at Cryos in the USA fully engaged with the recruitment process and subsequently passed all the screening tests and went on to have eggs frozen and available for use in MAR. This is considerably lower than almost all other studies in the literature we are aware of, although most of the previous studies reporting such data are now historic and pre-date the widespread introduction of egg freezing and are from egg donation programmes recruiting only anonymous (or predominantly anonymous) donors. They also pre-date the introduction of more rigorous screening requirements such as those published by the Practice Committee of the American Society for Reproductive Medicine in 2013 [5] and the Practice Committee of the Society for Assisted Reproductive Technology in 2021 [6].

For example, a study published in 1999 describing the outcome of 554 university students in Barcelona aged 18 to 25 who applied to be anonymous egg donors over a six-year period reported that 215 women (38.8% of initial applicants) were accepted but did not report how many of them went on to donate (which presumably would have been via fresh cycles given that egg freezing was not available at this time) [9]. By contrast, a study in Rockville, Maryland (USA) described that out of 7,310 women who applied to be donors between 2005 and 2007, only 303 women (4% of initial applicants) were successfully screened and went on to donate to a recipient [8]. Whilst the paper makes it clear that these women were donating anonymously, it is not clear whether their donations were used in fresh or frozen cycles. More recently, a study at a Public Gamete Bank in Portugal between 2011 and 2021 reported that of 466 women who applied to be donors, 26.18% of them were eventually accepted [15]. Since donor anonymity in Portugal was declared unconstitutional in May 2018 [16] and all donors recruited after this date had to agree to be ID-release, the data reported contains a mix of donor with different ID-choices as well as donors whose oocytes were eventually used in both fresh and frozen MAR. Therefore, to the best of our knowledge, our study of egg donor applicants at Cryos International in the USA is unique because it examines egg donor recruitment in a situation where all donated eggs were frozen, and the donor could choose whether to be ID-release or not.

In our previous study of sperm donor recruitment [10], we were able to compare data from Cryos sperm banks in both Denmark and the USA. However, since commercial egg banking was not allowed in Denmark (see [12]) we were unable to make a direct USA/Denmark comparison in this study. But, since ID-choice was an option for egg donor applicants in the USA, we were able to take this into account during our analysis. Somewhat surprisingly, unlike the situation with sperm donors at the same gamete bank [10], the final acceptance rate for egg donors did not differ between women who chose at the outset to be ID-release compared to those who did not (see Supplementary Table 1). Interestingly, as with the situation described for sperm donors [10], more accepted and released egg donors who began the process by selecting non-ID release subsequently opted to change to ID release (53.57%) compared to the other way around (9.09%) (see Supplementary Table 3). Again, we speculate whether this change of mind is a consequence of the counselling and general familiarity that will happen as the candidate egg donor starts engages with the recruitment process, but we cannot explain why it is notably higher than that seen in sperm donors during the same period at the same clinic (see Table III of [10]).

In comparison to the candidate sperm donor population described previously [10], none of the reasons that egg donors were lost from the recruitment pathway were related to their initial choice of ID-type (see Supplementary Table 2). The most common reasons for candidate egg donors being lost from the process was because they did not meet the selection criteria (e.g. age, BMI) as determined by the initial online screening questionnaire (51.17%) or they voluntarily withdrew or failed to respond, did not attend an appointment, or did not return a questionnaire (26.36%). The latter is a somewhat different profile from that described for sperm donors (see Table II of [10]), where 61.19% of men voluntarily withdrew or failed to respond during the recruitment process. Questionnaire data from the same clinic obtained in 2020 and 2023 shows that the motivations of sperm and egg donors were remarkably similar [17] and so cannot explain this difference. In the study of 7,310 women who applied to be oocyte donors in Rockville, Maryland (USA) a total of 41% dropped out before donating and this was higher in younger women (< 25 years of age) and those of African-American heritage [8]. Since we did not have any demographic information about the women included in our study, we are unable to conclude whether a similar relationship was observed in Florida. Similarly, of the 466 candidate egg donors studied in Portugal between 2011 and 2021, withdrawal or failure to attend were the main reasons for the exclusion of egg donor candidates [15]. In fact, a total of 73.8% of the Portuguese applicants were excluded which is higher than the 26.36% that we report in Florida in 2018 and 2019. However, since the authors provided no further details of this population, we are unable to conclude anything further from their study. Similarly, the study of university students in Barcelona [9] did not clearly report their drop-out rates.

We found that only 19.69% of candidate egg donors reported a disqualifying health condition or failed a screening test which prevented them from donating. This is surprisingly similar to the 17.41% found for sperm donors in the same clinic and during the same time-period [10] but is higher than some of the figures provided in other papers about egg donation, despite their different approaches and populations. For example, in the study of candidate egg donors in Rockville, Maryland (USA) [8], a total of 51 women were screened out for medical reasons (less than 1% of all applicants but 5.99% of those that passed the written application and medical questionnaire). In the population of University students from Barcelona [9] 10.5% of applicants were rejected because of previous family history or ‘personal pathologies’ (the paper did not separate the two), 7% were rejected because of gynaecological problems or ultrasonographic results, and 4.9% because of their blood test results. Interestingly no tests were done for Chlamydia trachomatis or cystic fibrosis because of the high costs involved. Unfortunately, the paper reporting on candidate donors in Portugal [15] did not describe the medical screening process of their donors in sufficient detail to facilitate a comparison to our data.

As with our paper on candidate sperm donors [10], a major strength of this study is the sheer number of candidate egg donors examined (*n* = 2,443) and the fact it covers the same time-period (2018 and 2019) in the same gamete bank with the same staff and IT system. This means that, as far as possible, the data of both sperm and egg donation is directly comparable and there is limited opportunity for variability in data collection which might influence the results. However, a similar limitation is that we only had anonymised summary data to work with, and we did not have any information about basic demographic characteristics of the egg donor applicants (e.g., ethnicity, age etc.) or any information about their lifestyle or medical history that may have been helpful when interpreting our findings.

## Conclusion

In conclusion, this study provides further evidence to illustrate how challenging egg donor recruitment processes are, with only a small fraction of those who initially apply ultimately being accepted and having samples certified as safe for use in treatment. It highlights that, unlike the situation previously described for candidate sperm donors [10], this is not influenced by the ID-release decisions made by the candidate donor at the outset. Given the investment of time and resources into the various stages of egg donor recruitment, it may be worthwhile to conduct further research to better understand why so many candidate egg donors are lost in the process. This could ultimately increase the number of donors recruited (through enhanced information, support, and reassurance during the recruitment processes), while maintaining the high standards of selection for safety and clinical effectiveness of the donations which are eventually released for use. This could ultimately reduce the financial cost of egg donor recruitment, and therefore lower the price to recipients, thus making it more affordable to those who are ineligible for state or insurance funded treatment.

## Supplementary Information

Supplementary Material 1.

## Data Availability

The data underlying this article were provided by Cryos International by permission. Data will be shared on request to the corresponding author with the permission of Cryos International.
