# Supplementary material for: An analysis of the outcome of 2,443 women applying to be donors at a commercial egg bank in the USA
Source: Reprod Biol Endocrinol. 2026 Jul 13;24:69. doi: 10.1186/s12958-026-01578-1 (PMC13362209; doi:10.1186/s12958-026-01578-1)
Supplement: Supplementary file 1 — Supplementary Material 1. [file 12958_2026_1578_MOESM1_ESM.docx]

**Supplementary Table 1** Proportion of egg donors that have samples frozen and available for use compared to those that initially apply, according to their initial identity-release preference.

|  | ID release | Non-ID release | Total |
| --- | --- | --- | --- |
| Total | 2.94%  (33/1122) | 2.12%  (28/1321) | 2.50%  (61/2443) |
|  | χ_2_ = 1.682; Df = 1; Z = 1.297; p = 0.1947 | |  |

ID: identity, Df: degrees of freedom

**Supplementary Table 2** Summary of reasons for candidate egg donor loss through the recruitment process, by initial identity-release choice

|  | **ID release** | **Non-ID release** | **Total** |
| --- | --- | --- | --- |
| Rejected at initial screening questionnaire: | | | |
| Total | 52.58%  (590/1122) | 49.96%  (660/1321) | 51.17%  (1250/2443) |
|  | χ_2_ = 1.670; Df = 1; Z = 1.292; p = 0.1963 | | |
| Applicant withdraws or fails to respond (at any stage): | | | |
| Total | 25.49%  (286/1122) | 27.10%  (358/1321) | 26.36%  (644/2443) |
|  | χ_2_ = 0.8106; Df = 1; Z = 0.9003; p = 0.3679 | | |
| Rejected owing to a health issue or because of failure of a genetic test or one or more tests for infectious diseases (at any stage): | | | |
| Total | 18.89%  (212/1122) | 20.36%  (269/1321) | 19.69%  (481/2443) |
|  | χ_2_ = 0.8274; Df = 1; Z = 0.9096; p = 0.3630 | | |
| Rejected because of poor ovarian response or poor egg quality: | | | |
| Total | 0.0%  (0/1122) | 0.23%  (3/1321) | 0.12%  (3/2443) |
|  | χ_2_ = 2.545; Df = 1; Z = 1.595; p = 0.1106 | | |

NB: Excluded from this analysis are 4 candidate donors (1 ID release and 3 Non-ID release) who were rejected at the nurse consultation stage for non-medical reasons.

**Supplementary** **Table 3** Proportion of egg donors that had eggs frozen and that decided to swap from non-identity release to identity release (or vice versa) during the donation process.

|  | ID release to non-ID release | Non-ID release to ID release | Total |
| --- | --- | --- | --- |
| Total | 9.09%  (3/33) | 53.57%  (15/28) | 29.51%  (18/61) |
|  | χ_2_ = 14.920; Df = 1; Z = 3.863; p <0.0001 | |  |

ID: identity, Df: degrees of freedom
